# THE PARADOXICAL EXPERIENCES OF INFORMAL CAREGIVERS DURING THE TRANSITION FROM HOME TO A NURSING HOME

**DOI:** 10.1093/geroni/igac059.085

**Published:** 2022-12-20

**Authors:** Lindsay Groenvynck, Bram de Boer, Audrey Beaulen, Erica De Vries, Jan Hamers, Theo van Achterberg, Judith Meijers, Hilde Verbeek

**Affiliations:** Maastricht University, Maastricht, Limburg, Netherlands; Maastricht University, Maastricht, Limburg, Netherlands; Maastricht University, Maastricht, Limburg, Netherlands; Maastricht University, Maastricht, Limburg, Netherlands; Maastricht University, Maastricht, Limburg, Netherlands; KU Leuven, Leuven, Vlaams-Brabant, Belgium; Maastricht University, Maastricht, Limburg, Netherlands; Maastricht University, Maastricht, Limburg, Netherlands

## Abstract

The transition from home to a nursing home is a complex and emotional process, especially for older persons with dementia. The informal caregiver usually plays a central role in this care process, which is often fragmented. Therefore, this study aims to analyze the experiences of informal caregivers of older persons with dementia during this transition.An interpretative phenomenological design was used to analyze secondary data. In-depth interviews were conducted with informal caregivers, in the Netherlands, between February 2018 and July 2018. The study identified three, interwoven paradoxes influenced by the healthcare system and the healthcare professionals providing care. The first paradox described the initial negative emotions related to a nursing home move. Those emotions are a paradox to the feelings of relief and acceptance later in the transition process. The second paradox was related to a prospective need to postpone the transition for as long as possible and a retrospective need for a timely transition plan. The third paradox defines an internal struggle for the informal caregivers of wanting to remain involved while simultaneously experiencing a need for distance from care responsibilities. This study identifies a fine line between optimal and fragmented transitional care. The results can motivate informal caregivers to start planning the move. Similarly, it allows healthcare professionals to provide tailored support. Future research should focus on defining these paradoxes and their link with the healthcare system to determine if the transition from home to a nursing home can be optimized.

